# Association of Protein and Vitamin D Intake With Biochemical Markers in Premature Osteopenic Infants: A Case-Control Study

**DOI:** 10.3389/fped.2020.546544

**Published:** 2020-11-24

**Authors:** Mohamed Mohamed, May Kamleh, Julia Muzzy, Sharon Groh-Wargo, Jalal Abu-Shaweesh

**Affiliations:** ^1^Department of Pediatrics, Sanford Children's Hospital, Fargo, ND, United States; ^2^Department of Pediatrics, University of North Dakota Grand Forks, Grand Forks, ND, United States; ^3^Department of Epidemiology, Harris County Public Health, Houston, TX, United States; ^4^School of Medicine, North Dakota State University, Fargo, ND, United States; ^5^School of Medicine, Case Western Reserve University, Cleveland, OH, United States; ^6^Department of Pediatrics, MetroHealth Medical Center, Cleveland, OH, United States; ^7^Department of Pediatrics, Cleveland Clinic, Cleveland, OH, United States

**Keywords:** vitamin D, neonatal, osteopenia, low birth weight (LBW) infant, nutrition, neonatal intensive care

## Abstract

Osteopenia in preterm infants (OP) remains an important challenge and is largely dependent on nutritional post-natal intake of factors influencing bone mineralization. We conducted a prospective case-control study to evaluate the importance of protein and vitamin D intake in OP among neonates with birth weight <1,250 g. Simultaneous serum parathyroid hormone (PTH), alkaline phosphatase (ALP), calcium (Ca), phosphorus (P), vitamin D and protein levels were measured during the first six post-natal weeks. At 6 weeks of age, OP was evaluated using wrist radiographs. Comparisons were analyzed using multivariate linear regression, receiver operating characteristic curves, χ2 and Wilcoxon Rank Sum. Of the 26 premature infants enrolled, 13 developed radiographic OP. Daily protein intake (coef = −0.40, *p* = 0.001) and vitamin D concentrations (21 ± 5.7 ng/ml) were significantly lower in the OP group compared to non-OP subjects. ALP concentration exceeding 619 IU/L, sensitivity of 76.9% and specificity of 75%, was predictive of OP at 6 weeks post-natally. PTH levels were higher at 6 weeks in OP subjects (193 ± 102.5 pg/ml, *p* < 0.001) compared to non-OP subjects. The findings in this study support the role of vitamin D and protein intake in the development of OP in VLBW infants and inform future practice and research on best practices for OP management.

## Introduction

In preterm infants, the risk of osteopenia of prematurity (OP), also known as neonatal rickets or metabolic bone disease (MBD), is a common and important concern (1). With medical advances that allowed for an increased survival of very low birth weight (VLBW) infants, the incidence of OP has increased (2, 3). Characterized by a reduction in bone mineral density (defined as the amount of minerals in bones), OP occurs in 20% to 30% of VLBW infants (4, 5) and is associated with multiple morbidities (6) including fractures (7), late onset respiratory distress (8), decreased linear growth (9) and dolichocephaly (10). The etiology of OP is multifactorial with risk factors including low gestational age (GA) and birth weight (BW), prolonged use of parenteral nutrition (PN), as well as exposure to corticosteroid and caffeine (3, 11, 12).

Optimizing early growth through nutritional interventions generates positive and lasting effects on bone growth and may partially counteract reduced bone mineral density in the preterm (13). The role of phosphorus (P) and calcium (Ca) intake has been well-established in the etiology of OP (14). Vitamin D also plays an important role in Ca homeostasis and bone mineralization during pregnancy and early extrauterine life (15). Inadequate vitamin D has been suggested as a cause of OP (16), however, several studies have reported normal levels of 25-hydroxy (25-OH) vitamin D in preterm infants with OP (17, 18). This inconsistency may be attributable to the fact that there is no current consensus on the recommended dose of vitamin D for prevention of OP. Currently, recommended guidelines of vitamin D dose for prevention of OP ranges between 200 and 1,000 IU/day (19–22). Thus, there is still a need to better characterize the influence of vitamin D on OP risk in VLBW infants.

Kuschel and Harding (23) conducted a systematic review and concluded that fortifying the nutrition of preterm babies with protein improved growth and bone mineral density. Protein is a major component of the osteoid bone matrix and adequate protein intake is vital for normal growth and development of healthy bone. However, the impact of protein intake on the development of OP has not been extensively studied.

Currently, there is no single reliable diagnostic technique to detect OP in premature infants (24). Therefore, there are no standard recommendations to screen for OP in preterm infants. Biochemical markers such as serum level of alkaline phosphatase (ALP), parathyroid hormone (PTH) and serum P have been previously used to screen for OP, but the value of these markers has been challenged (25). While some studies showed correlation between ALP or P levels and OP (14, 25–27), others failed to show this correlation (28–30). Wrist x-ray can also be used to diagnose OP; although bone mineralization needs to be decreased by at least 20–40% for these changes to be visible (31, 32).

The primary objective of this case-control study was to investigate how vitamin D and protein intake in the first 6 weeks of life influence development of radiological OP. Secondary objectives included investigating the role of Ca, P and PTH in OP risk and examining the predictability of ALP level to detect OP.

## Methods

### Study Center and Subjects

Infants who were admitted to the neonatal intensive care unit (NICU) at Rainbow Babies and Children's hospital were enrolled in this prospective case-control observational study. The study was approved by the Institutional Review Board at University Hospitals Case Medical Center (IRB98578) and informed consent was obtained from the parents before enrollment. Infants who were included in this study weighed <1,250 g and were born during the period of May 2009 to March 2010. Neonates who had major congenital abnormalities (i.e., major chromosomal abnormalities, inborn error of metabolism or lethal syndromes), suspected bone or muscular diseases (i.e., such as osteogenesis imperfect, hypophosphatasia, achondroplasia and congenital muscular dystrophy syndromes) and/or patients with direct bilirubin >2 were excluded.

### Study Design

This was a 10-month prospective observational study. All aspects of this study were part of the NICU's routine protocol, except measurement of PTH, vitamin D and wrist radiographs which were conducted as per the study's protocol. General feeding practices in our unit were as follows: Parenteral Nutrition (PN) usually started within 24 h after birth to provide glucose 5–10% solution to maintain the glucose infusion rate at 5–10 mg/Kg/min and protein at 1 gram/kg/day which is increased gradually to 3 grams/kg/day over few days. PN was discontinued when enteral feeding reached 100–120 ml/kg/day. Enteral feeding via nasogastric tube was gradually introduced according to the decision of the attending physician and advanced according to a birth weight (BW) advancement protocol. Human milk fortification was usually introduced when enteral feeding volume reached 80 to 100 ml/Kg/day. Hypocalcemia or hypophosphatemia levels were corrected with oral P or Ca supplementation based on judgement of managing neonatologist/dietitian. Oral multivitamins including vitamin D at 400 IU/day were added once enteral feeding reached 150 mL/kg/day. Days of PN, total parenteral nutrition (TPN) and “nothing by mouth” (NPO) were compared between OP and non-OP groups.

Daily intake of Ca, P, protein and vitamin D from enteral and parenteral sources were followed and recorded during the first 6 weeks of post-natal age. Serum levels of ALP, total Ca, and P were obtained weekly or biweekly as part of the routine clinical practice at our NICU for VLBW infants. Total protein intake was recorded from daily intake of protein from enteral feeds and TPN. Average daily protein intake (g/kg) was then calculated. Serum level of 25-OH vitamin D and PTH were obtained at 6 weeks of age as part of the study protocol. Infant history and diagnoses and total doses of medications known to be associated with OP (hydrochlorothiazide, furosemide, hydrocortisone and dexamethasone) were also collected for the first 6 weeks of life.

Serum PTH values were measured using the ADVIA Centaur® Intact PTH assay (Siemens Healthcare, Malvern, PA, USA). Vitamin D (25-OH) was measured using the LIAISON® 25-OH Vitamin D TOTAL assay which uses chemiluminescent immunoassay technology (33). ALP level was determined using the procedure published previously by Bowers and McComb (34).

Left wrist x-rays were performed for all study subjects at 6 weeks of life. We chose 6 weeks of age as our evaluation point to capture the earliest changes in bone mineralization. All radiographs were performed and evaluated by a single experienced pediatric radiologist who was masked to laboratory values and clinical diagnoses. Radiographs were classified according to the scoring of Koo et al. (31): grade 0: normal bones; grade 1: presence of bone rarefaction; grade 2: presence of bone rarefaction associated with metaphyseal alterations (fraying and cupping) and subperiosteal new bone formation and grade 3: the above changes with the presence of spontaneous fracture. Patients were classified into two groups: normal or abnormal wrist x-rays.

### Statistical Analysis

Descriptive statistical methods were used to describe the study population. Continuous data was analyzed using Wilcoxon Rank Sum test and categorical data was analyzed using χ2 analysis and Fisher exact test for small sample cross tables. Data are reported as mean ± standard deviation or median (range).

To examine the association between the development of OP and nutrient intake of Ca, P, protein and vitamin D, multivariable linear regression models for repeated measures were used to compare the profile of nutrient intake between the two outcome groups, adjusted for GA, gender and Apgar score at 5 min. Similar methods were used to compare blood serum levels between OP and non-OP groups. The correlation of serum ALP levels with x-ray results was determined by Spearman's correlation analysis. Receiver operating characteristic curve (ROC) and area under the curve was generated to compare the accuracy of ALP as a serologic marker for OP. Statistical analyses were performed using SAS 9.4® (SAS Institute, Cary, NC, USA) and a *p*-value of < 0.05 was used to indicate significance.

## Results

### Patient Characteristics

Twenty-six eligible premature infants with GA ranging from 23 to 31 weeks were enrolled into the study. The mean GA was 25.8 ± 0.7 and mean BW of 741 g. Twenty seven percent of patients were Caucasian, 65% were African American and 8% were Hispanic or Latino. Out of the 26 patients, 13 (50%) were identified with radiological evidence of OP at 6 weeks of age. No significant differences in the demographic characteristics of the infants were observed between OP and non-OP groups (Table 1).

**Table 1:** Demographic characteristics of preterm infants (*n* = 26) admitted to NICU with and without radiological evidence of osteopenia at 6 weeks of age.

**Variable**	**Mean (SD)**		*p*-value^*^
	**No Osteopenia** **(*n* = 13)**	**Osteopenia** **(*n* = 13)**	
Gender (male), *N* (%)	4 (30.77)	6 (46.15)	0.42
Gestational age (weeks)	25.9 (2.1)	24.1 (1.3)	0.15
Birth weight (grams)	764 (176)	718 (151)	0.49
Apgar score at 5 min, *N* (%)	4 (30.8)	5 (38.5)	0.68
Head circumference (cm)	23.2 (1.64)	22.4 (1.53)	0.19
Length (cm)	32.7 (3.36)	31.9 (2.60)	0.54

*The reported p-values were based on Fisher exact test for categorical variables and t-test for continuous ones, which were consistent with non-parametric tests in our cases. GA range: 23–31; Birth weight range: 500–1,100 (g)*.

* *Significant at p < 0.05*.

### Feeding Protocol

There was no significant difference in the median number of days on PN in the first six weeks and the median number of days to reach full feeding (120 ml/kg/day) between the two groups. However, infants who developed OP had a significantly higher median number of days being NPO, median (range): 11 (6–24) days vs. 6 (2–19) days, *p* = 0.03; for the OP and non-Op groups, respectively, Table 2.

**Table 2:** Association between Osteopenia and days of supplemental feeding among preterm infants (*n* = 26).

**Variable**	**No Osteopenia (*n* = 13)** Median^*^ (range)	**Osteopenia (*n* = 13)** Median^*^ (range)	*p*-value^**^
Days of NPO	6 (2–19)	11 (6–24)	0.03
Days to full feeding (120 ml/kg/day)	32 (22–39)	27 (11–41)	0.54
Days of PN intake	27 (13–42)	32 (22–42)	0.31

* *Median of the variables in the first 6 weeks of life. PN = parenteral nutrition*.

** *P-values based on the Wilcoxon Rank sum test. Significant at p < 0.05*.

Eleven (42.3%) of the patients received hydrochlorothiazide, 20 (76.9%) received furosemide, six (23.1%) received hydrocortisone and 10 (38.5%) received dexamethasone. Total administered medication dosages for each of the two groups is shown in Table 3. There was no significant difference in total dose of hydrochlorothiazide, furosemide, hydrocortisone or dexamethasone administrated during the first 6 weeks of life between the two groups.

**Table 3:** Dosage of administered medications in the first 6 weeks of life by osteopenia status.

**Medication**	**Total dosage (mg/kg/week)**	
	**Osteopenia**	**Non-osteopenia**
Furosemide	52.1	49
Dexamethasone	10.42	8.16
Hydrocortisone	9.9	12.6
Hydrochlorothiazide	886	740

### Daily Intake

Daily intake (unit/kg) data were compared between OP and non-OP groups using mixed-effect linear regression modeling for repeated measures data adjusting for GA, gender and Apgar score at 5 min. The results are shown in Tables 4A,B.

**Table 4A:** Co-variate adjusted multi-variable linear regression results^a^ of the association between protein intake^b^ and osteopenia risk among preterm infants (*n* = 26).

**Outcomes**	**Covariates**	**Estimate (error)**	*p*-value^*^
Total protein intake (unit/kg)	Osteopenia	−0.4 (0.12)	0.001
	Day of life	0.01(0.004)	0.004
Parenteral nutrition protein intake (unit/kg)	Osteopenia	0.43(0.17)	0.01
	Day of life	−0.086(0.007)	<0.0001
Enteral protein intake (unit/kg)	Osteopenia	−0.92(0.16)	<0.0001
	Day of life	0.10 (0.007)	<0.0001

a *Multi-level linear regression models were adjusted for GA, Apgar score, and gender. The final model was determined via backward selection procedures*.

* *Significant at p < 0.05*.

b *Daily protein intake (unit/kg) until 6 weeks post-natally*.

**Table 4B:** Co-variate adjusted multi-variable linear regression results^a^ of the association between nutrient intake^b^ and osteopenia risk among preterm infants (*n* = 26).

**Outcomes**	**Covariates**	**Estimate**	*p*-value^*^
Vitamin D intake (unit/kg)	Osteopenia	−540.2(103.8)	<0.001
	Day of life	16.0(2.93)	<0.0001
Calcium intake (unit/kg)	Osteopenia	−23.6(19.3)	0.22
	Day of life	5.14(0.56)	<0.0001
Phosphorus intake(unit/kg)	Osteopenia	−14.9(11.04)	0.18
	Day of life	2.85(0.32)	<0.0001

a *Multi-level linear regression models for repeated measures were adjusted for GA, Apgar score, and gender. The final model listed in the table was determined by backward selection procedures*.

* *Significant at p < 0.05*.

b *Daily nutrient intake (unit/kg) in last 2 weeks prior to X-ray diagnosis (at 42 day of life)*.

Daily protein intake (gram/kg) was significantly lower (coef = −0.40, *p* = 0.001) in the OP group. The protein intake from an enteral source was lower (coef = −0.92, *p* < 0.0001), while the one from parenteral source was higher (coef = 0.43, *p* = 0.01) in the OP group. Similarly, the daily vitamin D intake (IU/kg per day) in the last 2 weeks before the diagnosis (4–6 weeks of life) in the OP group was also significantly lower (coef = −540, *p* < 0.001) when compared to the non-OP group. However, no significant differences in daily Ca and P intake between the two groups were observed (Table 4B).

### Serum Levels

**Figure 1:**
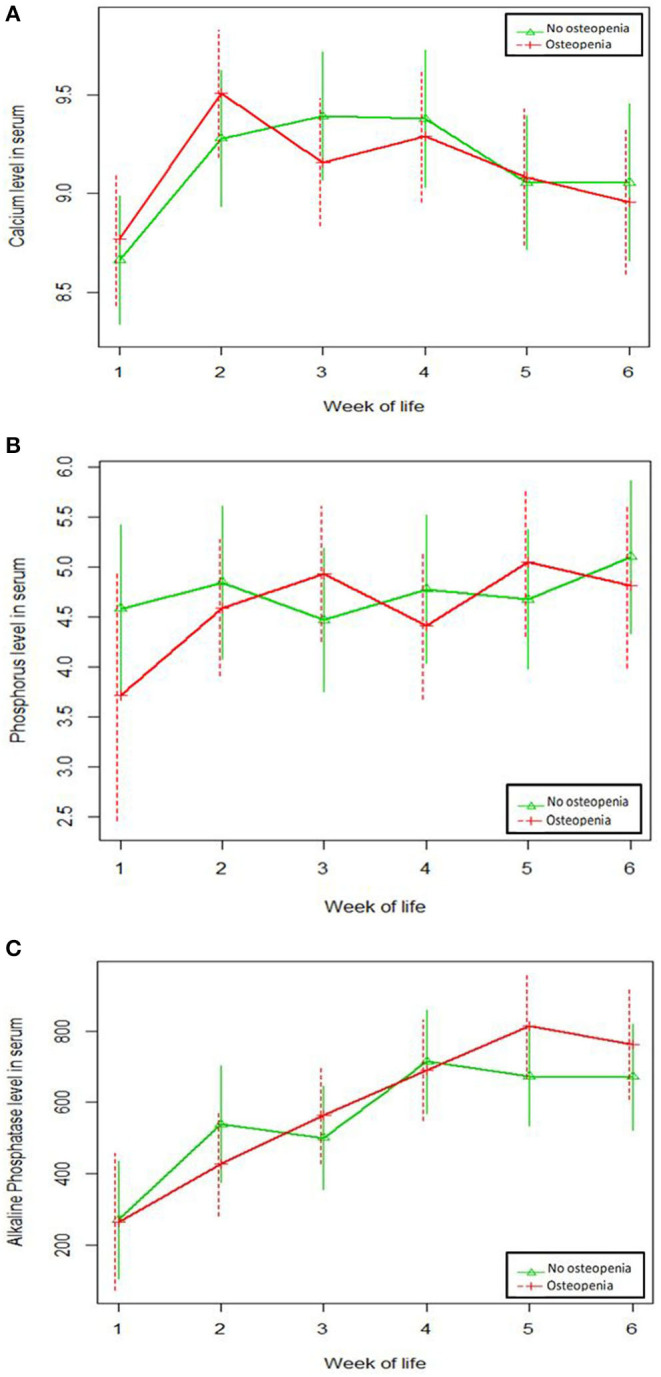
No significant differences in serum levels in the first 6 weeks of life between osteopenia and non-osteopenia groups was observed for **(A)** calcium levels; **(B)** phosphorus levels; **(C)** alkaline phosphatase levels.

**Figure 2:**
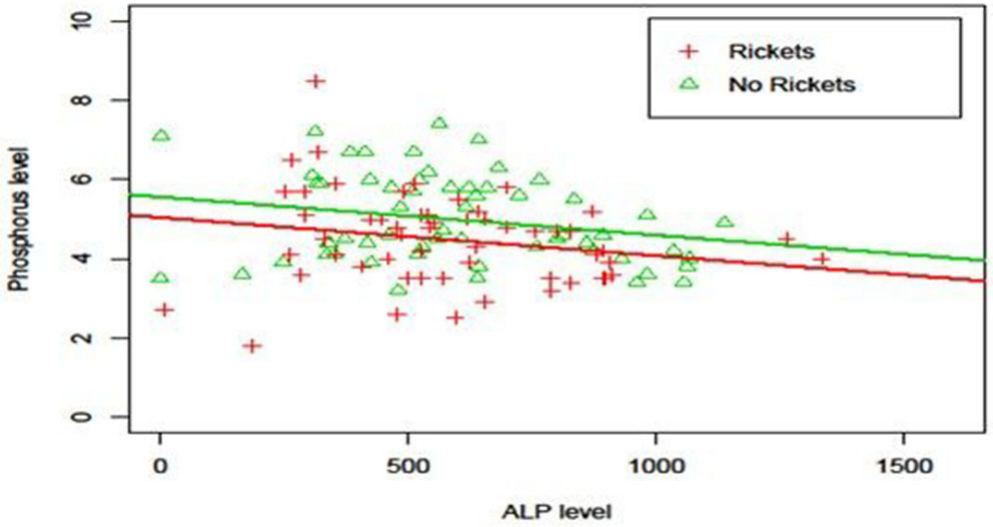
Relationship between ALP level and Phosphorus level. Phosphorus levels decreased significantly (*p* = 0.01) with respect to ALP level (Phosphorus = 5.73 −0.001xAlkPhos−0.59xRickets) and, at each ALP level, phosphorus level was significantly higher (*p* = 0.005) in the non-osteopenia group than that in osteopenia group.

**Figure 3:**
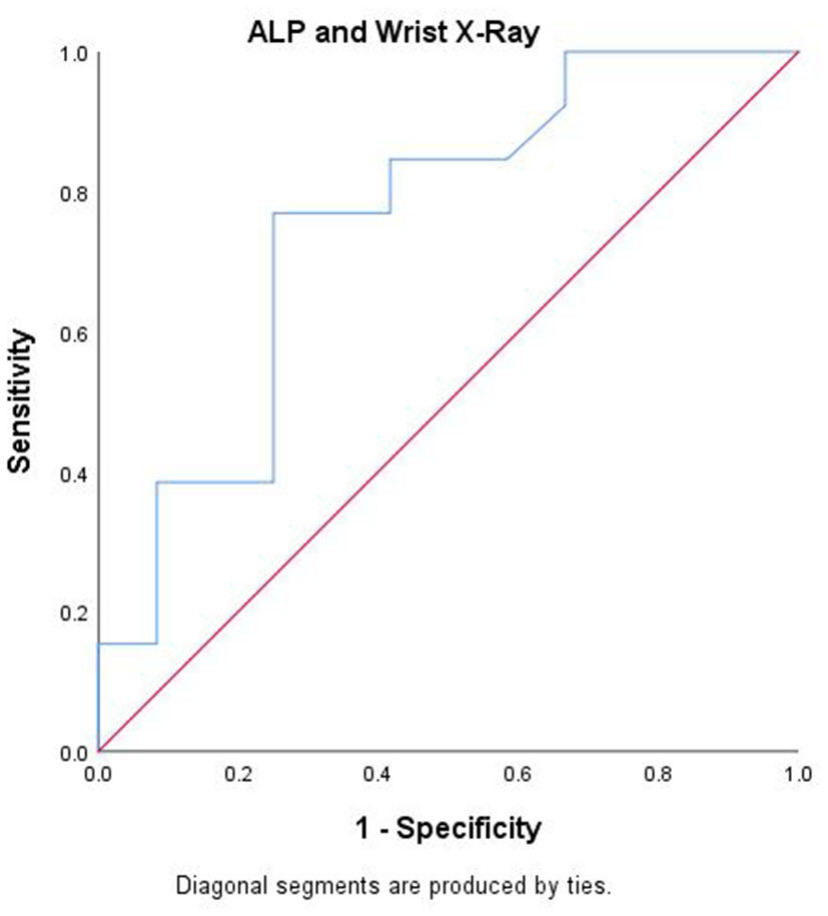
Receiver-operating characteristic curve (ROC) analysis for alkaline phosphatase in detecting osteopenia at week 6 post-natally.

There were no significant differences in serial serum levels of Ca or P or ALP in the first 6 weeks of life between the two groups (Figures 1A–C). ALP was found to have a significant negative correlation with P serum level (Figure 2), however, there was no significant relation between serum ALP and Ca levels. GA was significantly inversely correlated with ALP levels (ρ = −0.68, *p* = 0.003). Table 5 and Figure 3 show the ROC analysis of ALP values for the diagnosis of OP. The optimal cutoff point for ALP was 619 IU/L, where sensitivity was found to be 76.9% and specificity was 75%. The area under the curve is 0.753, denoting there is a 75% chance that ALP can identify OP cases when compared to wrist x-ray results in our sample.

**Table 5:** ROC analysis for alkaline phosphatase levels and wrist x-ray results at 6 weeks.

Area under the ROC curve	0.753
Standard error	0.10
95% Confidence Interval	0.557–0.949
*p*-value^*^	0.032

*ROC, receiver operating characteristic curve*.

* *Significant at p < 0.05*.

Vitamin D (25-OH) concentration was lower (*p* < 0.001) at 6 weeks chronological age in infants with radiologic diagnosis of OP (21 ± 5.7 ng/ml), compared to infants without radiological evidence of OP (39.3 ± 5.7 ng/ml). PTH serum level was higher (*p* < 0.001) at 6 weeks chronological age in infants with radiologic diagnosis of OP (193 ± 102.5 pg/ml), compared to infants without radiological evidence of OP (58.7 ± 49.7 pg/ml).

## Discussion

Despite improvements in nutritional management, OP continues to be among the most common morbidities affecting extremely premature infants. Furthermore, there is no unified diagnostic method to identify OP. Many factors have been linked to the development of OP. To the best of our knowledge, this is the first study to document a relationship between daily intake of protein and vitamin D and the development of OP in preterm infants with a birth weight of <1,250 grams.

Previous studies have shown that vitamin D intake as low as 200 IU/day may have the same effect in preventing OP as do higher doses. Backstrom et al. (35) found no difference in the incidence of MBD at 3- and 6-months between infants born <33 weeks gestation who received either low or high dose vitamin D supplements, even after correcting for potential risk factors. Similarly, McIntosh et al. (36) found no correlation between 25 hydroxy-vitamin D serum concentrations and radiographic appearances of MBD. Koo et al. (17) evaluated supplementation with three dose regimens (200, 400, and 800 IU/day) of vitamin D in 71 preterm infants and found that serum vitamin D concentrations were similar between infants with radiological rickets and those without. In our study, however, we found significantly lower 25-OH vitamin D concentrations (unit/kg) in infants with radiological OP. The mean 25-OH vitamin D level in infants without OP was 39.3 ± 5.7 ng/mg, which is close to the current AAP guidelines to achieve serum 25(OH)D status of at least 50 nmol/L (37). To the best of our knowledge, our study was the first to examine the effect of vitamin D intake per body weight on OP risk among neonates.

Research investigating the association between serial protein intake and the risk of OP in preterm infants is limited. In this study, we showed that higher daily protein intake reduced the risk of OP. This is consistent with Viswanathan et al. (38), in which VLBW infants who had lower intake of protein during the first 8 weeks of life were more likely to develop MBD (38). Since human milk provides inadequate protein and mineral content to VLBW infants, fortifying breast milk has been proposed to meet the nutritional needs of the preterm infants. Our results show that protein-fortification is also essential in reducing the risk of OP. It is important to note that while protein intake ranged from 2.2 to 3.6 g/kg/day among our study subjects, the current recommended daily protein intake has increased to 3.5–4 g/kg/day (39). Further studies are clearly needed to evaluate the ability of higher daily protein intake to prevent the development of OP in VLBW infants.

As major inorganic constituents of bone, the role of nutritional Ca and P deficiencies in the etiology of bone disease is well-established (2, 3, 14, 38). In our study, Ca and P daily intake were similar in the two groups. Furthermore, serum levels of these two minerals during the first 6 weeks post-natally were not statistically different between osteopenic and non-osteopenic patients. We suspect this finding may be due to aggressive Ca and P supplementation in these high-risk infants. Our results are in agreement with So and Ng (40) who reported normal Ca level in osteopenic infants. Similarly, Hung et al. (25) reported similar levels of Ca concentration in osteopenic premature infants (≤34 weeks' gestation) compared to those with no evidence of OP. In a recent review of diagnostic factors related to MBD of prematurity, Faienza et al. (40) concluded that the assessment of serum calcium levels is not a reliable screening tool for OP because newborns can maintain normal calcium values despite bone Ca loss. Other investigators also challenged the association between bone mineral content and mean serum P levels in preterm infants (28). Abdallah et al. (24) reported no significant differences in serum P levels between OP and non-OP preterm infants. Still, a number of studies have proposed that lower plasma phosphate levels correlated with increased OP risk in VLBW infants (41, 42). In a systematic review by Rehman and Narchi (3), it was suggested that serum P levels <1.8 mmol/L was associated with the presence of radiological evidence of OP and thus P supplementation should be started. In another study, Harrison and Gibson (43) reported that preterm infants with low serum inorganic phosphate (<2 mmol/L) are at increased risk of OP. They proposed that the use of serum phosphate levels in combination with ALP levels can increase the sensitivity of screening and identification of infants at risk of MBD. We don't believe that this data is contradictory. In fact our data indicate that OP can still develop in VLBW infants despite adequate Ca and P intake and normal serum levels. It also highlights the importance of other factors to be considered for prevention and treatment of babies with OP including protein and Vitamin D intake.

Serum ALP is a known marker of bone turnover (44). In this study, we found no significant differences in ALP levels (mean and peak) between infants with and without OP. A plausible explanation for this finding may be that the small sample size of our study did not result in enough statistical power to detect a difference. However, other studies have reported similar findings. Mitchell et al. (30) noted mean peak ALP levels exceeded 600 IU/L in both osteopenic and non-osteopenic VLBW patients with no significant difference in peak ALP between the two groups. Similarly, Faerk et al. (28) also failed to find any association between ALP and OP in preterm infants (<32 weeks gestation) using dual energy x-ray absorptiometry (DEXA) scan. They showed that there was no significant difference in OP between infants with the highest (>1,200 U/l) and lowest (<600 U/l) measurements, suggesting ALP was not useful diagnostic or screening tool. In our study, although there were no differences in ALP levels between the two groups, when we compared wrist x-ray results to ALP serum levels at 6 weeks post-natally, the optimum cutoff value was 619 IU /L yielding a diagnostic sensitivity of 76.9% and specificity of 75%. This is comparable to Mitchell et al. (30), where in one review of 100 extremely low birth weight infants, they reported that ALP levels of >600 IU/L were commonly detected. However, no single value of ALP could be determined as predictive of the bone changes. ALP has been routinely used as a biochemical marker to screen for OP in preterm infants, however the predictability of the test is controversial with variation in the cut-off value (14, 24–27, 35). A total ALP level of above 750 IU/L has been shown to be suggestive of severe OP and may precede radiographical evidence of rickets in preterm infants (45). Backstrom et al. (35) suggested that serum ALP levels higher than 900 IU/L associated with a serum phosphate level lower than 1.8 mmol/l have a diagnostic sensitivity of 100% and specificity of 70%. Overall, our results indicate that ALP may not be an adequate diagnostic tool for OP secondary to overlap of ALP levels between the two groups and the fact that radiographic evidence of OP occurred in infants with ALP < 600 IU/L.

ALP values were also inversely correlated with gestational age. The risk of OP is known to declines (24) with advancing gestational age as bone mineral density increases. Since ALP is an early marker of osteoblast differentiation, increased serum ALP is a hallmark of a reduction in bone mineralization (46), which is expected with lower gestational age. A notable finding in our study, however, is the negative association between serum P levels and ALP, which has not been previously reported in OP literature. While P and ALP levels were not adequate diagnostic tools for OP in our study, this finding suggests a relationship between the two markers. A more comprehensive diagnostic strategy that includes all known biomarkers of MBD, including the ones identified in our study, may constitute a more useful approach than individual markers.

Serum PTH level has been proposed as an early marker in the monitoring and management of OP (47, 48). In our study, PTH levels were significantly elevated in OP patients despite similar serum Ca and phosphate levels between the two groups. Consistent with other findings that showed serum PTH levels >100 pg/ml may be indicative of MBD risk in VLBW infants (40), mean PTH serum level in our study among osteopenic neonates was 193 ng/ml. In a retrospective case series investigating osteopenic neonates (<1,000 g), Moreira et al. (49) concluded that elevated PTH serum levels may be a useful marker in identifying low birth weight neonates at risk for MBD. In a subsequent prospective study, Moreira et al. (48) suggested the sensitivity of PTH as an early diagnostic marker for MBD is higher than that of ALP, a conclusion also reported by Dokos et al. (50). Future studies should attempt to correlate PTH values with Ca and P levels as well as newer imaging techniques, such as quantitative ultrasonography and DEXA.

To the best of our knowledge, this is one of few studies that looked prospectively at potential risk factors for OP. The lack of prospective research in this area has been highlighted; this study adds to our current understanding of early life biomarkers of MBD of prematurity. Nonetheless, our study is not without important limitations. The main limitation is the small sample size and the fact that this is a single center study. Our findings might have been related to the unit's protocols and guidelines as well as provider variations that might not be applicable to other units. Further research including multiple sites may improve generalizability of study results. Use of wrist radiographs for the detection of OP might also be another limitation. To be detected by x-ray, at least 20% of demineralization needs to occur. We chose to use this method for its clinical feasibility and applicability. Future studies using quantitative ultrasounds and bone density measurements might be more accurate in diagnosis and correlation of factors associated with MBD of prematurity. Finally, our data doesn't include information that reflects the health status of the mother, which might have contributed to bone metabolism of premature neonates.

In conclusion, presently our understanding of the development of OP in premature neonates is limited and inconclusive. The findings in this study add to the existing literature, supporting the role of vitamin D and protein intake in OP risk of premature infants. Preterm infants with elevated levels of PTH are also at higher risk of developing OP and this might be helpful as a marker in at risk infants. The evidence on the role of Ca, P, and ALP levels in osteopenic neonates remains conflicting. Our data suggest an ALP optimum cutoff value of 619 IU /L, yielding a diagnostic sensitivity of 76.9% and specificity of 75%. This study helps to inform research and clinical practice toward modifying the nutritional management of VLBW infants in order to reduce OP risk. Clearer identification of risk factors and refinement of biomarkers for bone health in neonates is necessary and will enable earlier preventive strategies.

## Data Availability Statement

The datasets presented in this article are not readily available because Confidential information. Requests to access the datasets should be directed to mohamed.mohamed@sanfordhealth.org.

## Ethics Statement

The studies involving human participants were reviewed and approved by The study was approved by the Institutional Review Board at University Hospitals Case Medical Center. Written informed consent to participate in this study was provided by the participants' legal guardian/next of kin.

## Author Contributions

MM and JA-S: conceptualization. MM, JA-S, and SG-W: methodology. MK: formal analysis. MM: investigation. JM: data curation. MM and MK: writing–original draft preparation. MK, MM, SG-W, and JA-S: writing–review and editing. JA-S and SG-W: supervision. All authors contributed to the article and approved the submitted version.

## Conflict of Interest

The authors declare that the research was conducted in the absence of any commercial or financial relationships that could be construed as a potential conflict of interest.
